# Grammatical aspect and temporal distance in motion descriptions

**DOI:** 10.3389/fpsyg.2013.00337

**Published:** 2013-07-01

**Authors:** Sarah E. Anderson, Teenie Matlock, Michael Spivey

**Affiliations:** ^1^Department of Psychology, University of CincinnatiCincinnati, OH, USA; ^2^Department of Cognitive and Information Sciences, University of California, MercedMerced, CA, USA

**Keywords:** embodied cognition, mouse-tracking, grammatical aspect, motion verbs

## Abstract

Grammatical aspect is known to shape event understanding. However, little is known about how it interacts with other important temporal information, such as recent and distant past. The current work uses computer-mouse tracking (Spivey et al., 2005) to explore the interaction of aspect and temporal context. Participants in our experiment listened to past motion event descriptions that varied according to aspect (simple past, past progressive) and temporal distance (recent past, distant past) while viewing scenes with paths and implied destinations. Participants used a computer mouse to place characters into the scene to match event descriptions. Our results indicated that aspect and temporal context interact in interesting ways. When aspect placed emphasis on the ongoing details of the event and the temporal context was recent (thus, making fine details available in memory), this match between conditions elicited smoother and faster computer mouse movements than when conditions mismatched. Likewise, when aspect placed emphasis on the less-detailed end state of the event and temporal context was in the distant past (thus making fine details less available), this match between conditions also elicited smoother and faster computer mouse movements.

Everyday conversation is replete with reports of when and how events have occurred in the past. Take the sentences, “Last week David walked to the school,” and “Last week David was walking to the school.” Both sentences describe past events, but the former, marked with the simple past (verb+ed), focuses on completion of the event, and the latter, marked with past progressive (was verb+ing), on its ongoing nature. How does grammatical information influence our understanding of events, especially the reporting of past events? How does it interact with information about when an event has occurred, specifically, recent past vs. distant past? Here, we use a mouse-tracking task to explore how grammatical aspect and tense interact in perceptual simulations influence the comprehension of event descriptions.

Over the past several decades, linguistics research has significantly advanced our understanding of aspect and of how it works in various languages. One valuable observation is that languages often make a distinction between imperfective and perfective aspect. Simply stated, imperfective emphasizes the ongoing nature of an event, and perfective, the completion of an event (see Comrie, 1976). In some cases, this difference is realized through grammatical information, and in others, lexical information (see Croft, 2012, for discussion). In English, imperfective aspect is realized by using the past progressive verb form, as in *was walking*, and perfective aspect, by using the simple past verb form, as in *walked* (see Brinton, 1988). Another valuable observation from linguistics is that imperfective aspect gives the speaker and listener an internal view of event descriptions, at least more than perfective aspect does (see Langacker, 1987). A statement such as “Yesterday David was chopping wood” gives access to details about the event as it unfolds in time, including for instance, lifting the ax, slamming it into the wood, standing back to cut another piece of wood, lifting the ax again, and a simple past statement such as “Yesterday David chopped wood” focuses on the endpoint of the event or gives a diffuse sense of the entire event. Despite a wealth of information on aspect, including useful insights on crosslinguistic patterns and historical work, relatively little is known about how it is processed in everyday language, including how it influences the interpretation of when and how events occurred in the past.

In recent years, language theorists have begun to explore the role of aspect in processing everyday language. In a series of offline studies, Matlock (2011) found that varying aspectual information in event descriptions leads to consistent differences in how action is conceptualized. In one experiment, participants completed a sentence that began with a past progressive adverbial clause, “When John was walking to school,” or a simple past adverbial clause, “When John walked to school.” The results showed that participants mentioned more actions when completing sentences with past progressive adverbial clauses. In another experiment, participants read simple transitive sentences that implied a state change in objects, specifically, simple past “John painted houses last summer” or past progressive “John was painting houses last summer,” and then estimated how many houses had been painted. Their estimates were reliably higher with the progressive form (e.g., “was painting”). In related work by Matlock (2010) participants read the sentences “Bob planted pine trees along his driveway last week” or “Bob was planting pine trees along his driveway last week,” and then estimated the length of the driveway. Their estimates were reliably larger with past progressive. Together these results suggest that the past progressive leads to inferences about more action in a given time period than does the simple past. [For similar work on how aspect can influence attitudes about political candidates and political issues, see Fausey and Matlock (2010)].

Earlier research, specifically, on aspect in narrative comprehension showed compatible results. Madden and Zwann (2003) conducted several experiments that incorporated event descriptions with pictures to investigate how aspectual cues shapes the construction of situation models (see Zwaan and Radvansky, 1998, for discussion of situation models). They were especially interested in perfective and imperfective aspect (corresponding to English simple past and past progressive). In one experiment, participants viewed pictures of events that appeared to have just been completed or in progress, for instance, a car that had just gone through an intersection, or a car going through an intersection. Next they indicated whether sentences such as “The car sped through the intersection” (simple past) or “The car was speeding through the intersection” (past progressive) matched the scene depicted in the picture. Participants were found to be notably faster to read simple past sentences after having viewed depictions of completed events, but about equally fast after to read past progressive sentences after having viewed depictions of intermediate events. These results were consistent with another experiment in which participants read sentences and then made a speeded decision about whether pictures matched. Madden and Zwann (2003) offered various explanations for why there was no difference with the progressive form, including the possibility that readers perceptually simulated the actions at different stages of completion. For instance, people may have thought about the car being in different locations with “The car was speeding through the intersection” (e.g., entering intersection, in the middle of intersection, exiting intersection). Together, their results provided groundbreaking insights on how aspectual cues constrain the construction of situation models. Different aspectual cues were shown to yield real time processing differences with event descriptions (for similar findings, see also Morrow, 1985; Magliano and Schleich, 2000; Ferretti et al., 2007; Madden and Therriault, 2009; Bergen and Wheeler, 2010).

Additional work on the role of aspect in the time course of processing event descriptions has employed eye tracking. A recent study by Huette et al. (2012) used the blank visual world approach to explore how aspect would influence eye movements during the course of comprehending event descriptions without visual input (see Spivey and Geng, 2001, for information on blank visual world approach). In their study, participants listened to short descriptions of actions that included simple past or past progressive verb forms while they simply looked ahead at a blank screen. The results showed fewer eye movements and longer fixations on the blank screen with past progressive descriptions than with simple past descriptions, suggesting that participants conceptualized more action with the past progressive [consistent with Matlock (2011)].

The studies mentioned above resonate to contemporary theories of perception and action, and more specifically, to perceptual simulations. In this general view, it is assumed that cognitive abilities are grounded in sensorimotor experiences and that high-level processes are intimately linked to low-level processes (see Thelen and Smith, 1994; Barsalou, 1999; Zwaan, 2004; Gallese and Lakoff, 2005; Calvo-Merino et al., 2006; Gibbs, 2006). In the realm of language comprehension, nouns, verbs, adjectives, and other lexical information partially reactivates the actual perceptual or motor correlates of those constituents. In particular, comprehending an action description partially reactivates neural correlates associated with performing that action (see Pulvermuller, 2001; Hauk and Pulvermuller, 2004; Hauk et al., 2004), which in turn influences subsequent and current behavioral responses that rely on those same neural substrates (Glenberg and Kaschak, 2002; Boulenger et al., 2006; Nazir et al., 2007).

Given that grammatical aspect influences the way events are conceptualized, it certainly has the potential to influence the way goal-directed motion events are realized in real time. The past progressive form (*was* verb+ing) in English gives an internal perspective that highlights the moment-to-moment unfolding of an event, and the simple past (verb+ed), an external perspective that focuses on the end state of an event or provides a “snapshot” of the whole event (see Comrie, 1976; Langacker, 1987; Madden and Zwann, 2003). In the current work we investigate how these two forms influence the understanding of past events in which a mover traverses a path toward a goal. We use computer-mouse tracking (Spivey et al., 2005) to explore motor output in response to variations in aspect and temporal distance in motion descriptions. Earlier work with this approach discovered that mouse movements that accompanied past progressive motion descriptions resulted in longer durations than did mouse movements that accompanied simple past motion descriptions (Anderson et al., 2008). Here, we extend our approach to investigate aspect (past progressive vs. simple past) in the context of temporal distance (recent vs. distant past). Of interest is how these aspectual cues will play out with recent and distant past contexts. Social psychology research on construal-level theory has shown that temporally distant events are construed as relatively more abstract than temporally close events (Trope and Liberman, 2003, 2010; Liberman and Trope, 2008). We anticipate that events in the distant past may be simulated with a different perceptual character (less detailed, more punctate, with emphasis on the end state) than events in the recent past (more detailed, with more emphasis on the interstitial components of the event as it unfolds).

## Method

### Participants

Sixty-four undergraduates at University of Cincinnati participated for class credit in Introduction to Psychology courses. All were right-handed native speakers of American English.

### Materials and procedures

#### Verbal stimuli

Stimuli included 16 sentences about a person moving along a path. Each sentence had four variants realized by combining timeframe distance and aspect. Each represented an experimental condition, as shown in Table 1: recent past simple past; recent past progressive; distant simple past; distant past progressive.

**Table 1:** **Examples of target verbal stimuli that accompanied visual scenes**.

	**Simple past sentences**	**Past progressive sentences**
Recent past temporal description	Yesterday David walked to the university.	Yesterday David was walking to the university.
	Yesterday Paul ran to the lake.	Yesterday Paul was running to the lake.
	Yesterday Eric walked to the fairgrounds.	Yesterday Eric was walking to the fairgrounds.
Distant past temporal description	Last year David walked to the university.	Last year David was walking to the university.
	Last year Paul ran to the lake.	Last year Paul was running to the lake.
	Last year Eric walked to the fairgrounds.	Last year Eric was walking to the fairgrounds.

All sentences were read by a native speaker of American English and recorded using a Mac-based sound software. Each of the 16 experimental items was spliced to produce each of the four experimental conditions, ensuring that the prosody of the targets was otherwise identical. An additional 15 s of silence was added to the end of each target sentence, allowing us to time lock participants' mouse-movements to the raw time stamp of the sound files. The experimental items were counterbalanced across four presentation lists. Each list contained four instances of each condition, so a participant heard all the target sentence frames, but only one version of each.

#### Visual stimuli

**Figure 1:**
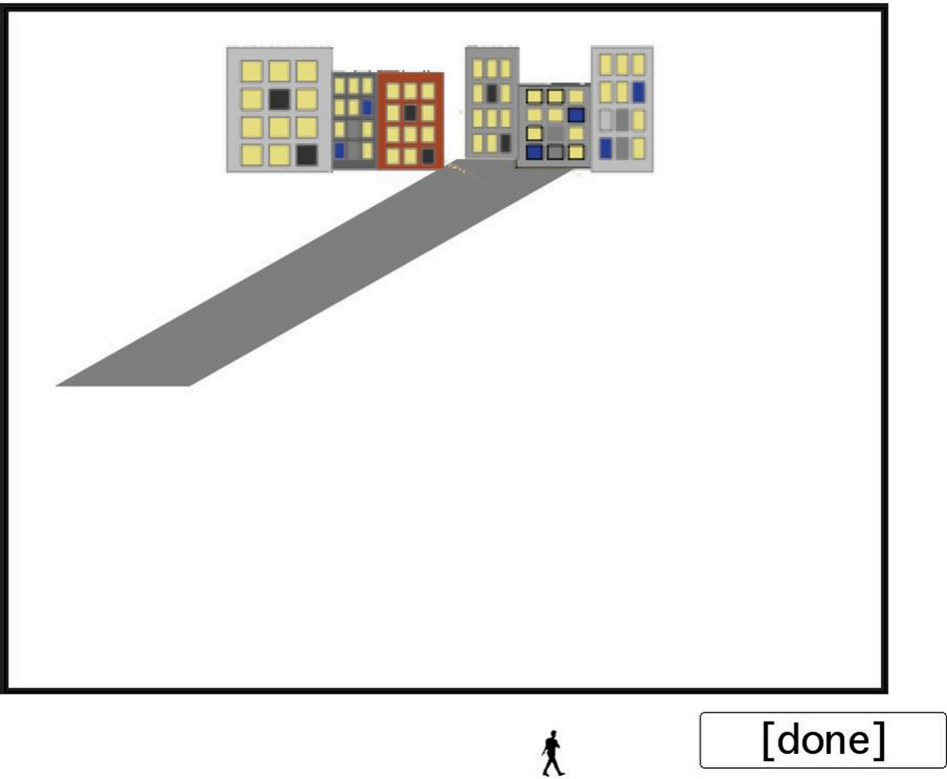
**Visual scenes like this one accompanied target sentences.** The silhouette figure at the bottom is the character in its starting position.

Corresponding visual scenes were created for each target sentence pair. Each target visual scene consisted of a diagonal path starting halfway up and on the far left side of the screen. The path slanted to the right, terminating at the middle top of the screen. A character was located to the right of the beginning of the path and under the destination, and separated from the scene by a black box that framed the destination and path, as shown in Figure 1. Items in the scene were created by hand or taken from clipart and edited in Adobe Photoshop. The only moveable item was the character, which subtended an average of 1.53° of visual angle in width by 2.05° in height. The destinations were an average of 11.22° in width by 4.09° in height, and the path itself occupied a square of 8.42° in width by 6.11° in height. The character was located 14.25° from the destination. The stimuli were presented using Macromedia Director MX, and mouse movements were recorded at an average sampling rate of 40 Hz. The display resolution was set to 1024 × 768.

Sixteen filler items were created to keep participants from developing strategies specific to the experimental sentences. Similar to the target sentences, all filler sentences began with a timeframe description. These filler sentences also included past progressive or simple past aspectual information, and conveyed movement (e.g., “Last month, Janet swam in the pool”) but not along the path. These were accompanied by 16 filler scenes, which had a short path beginning on the right side of the screen and slanting to the top, center of the screen.

### Procedure

Participants were first asked to make themselves comfortable in front of the computer and allowed to adjust the mouse and mouse-pad to a location that suited them. Participants then read the instructions, which asked them to place the character into the scene to make the scene match the sentence they heard. After indicating that they understood the task, participants were next presented with two practice trials, followed by the experimental task. At the onset of each trail, participants were presented with the entire visual scene. The sound file began after a 500 ms preview. Also, a “Done” button was present in the bottom left corner of the screen from the beginning of the trail. When participants were finished placing the character in the scene, they clicked on “Done” to move to the next trial. A blank screen with a button in the center labeled “Click here to go on” separated the trials. The entire experiment lasted about 10 min.

## Results

Mouse movements were recorded during the grab-click, transferal, and drop-click of the character in all experimental trials. Data from three participants who immediately clicked the “done” button for every trial (and thus, produced no mouse trajectories) were removed from analyses. There were no significant differences in movement onset latencies, suggesting that sentences from the different conditions were approximately equally understandable and acceptable. Above and beyond such simple reaction time measures, computer-mouse tracking is robust for measuring various indices of response and motor dynamics (Spivey et al., 2005). We investigated four of these indices here.

### Drop locations

**Figure 2:**
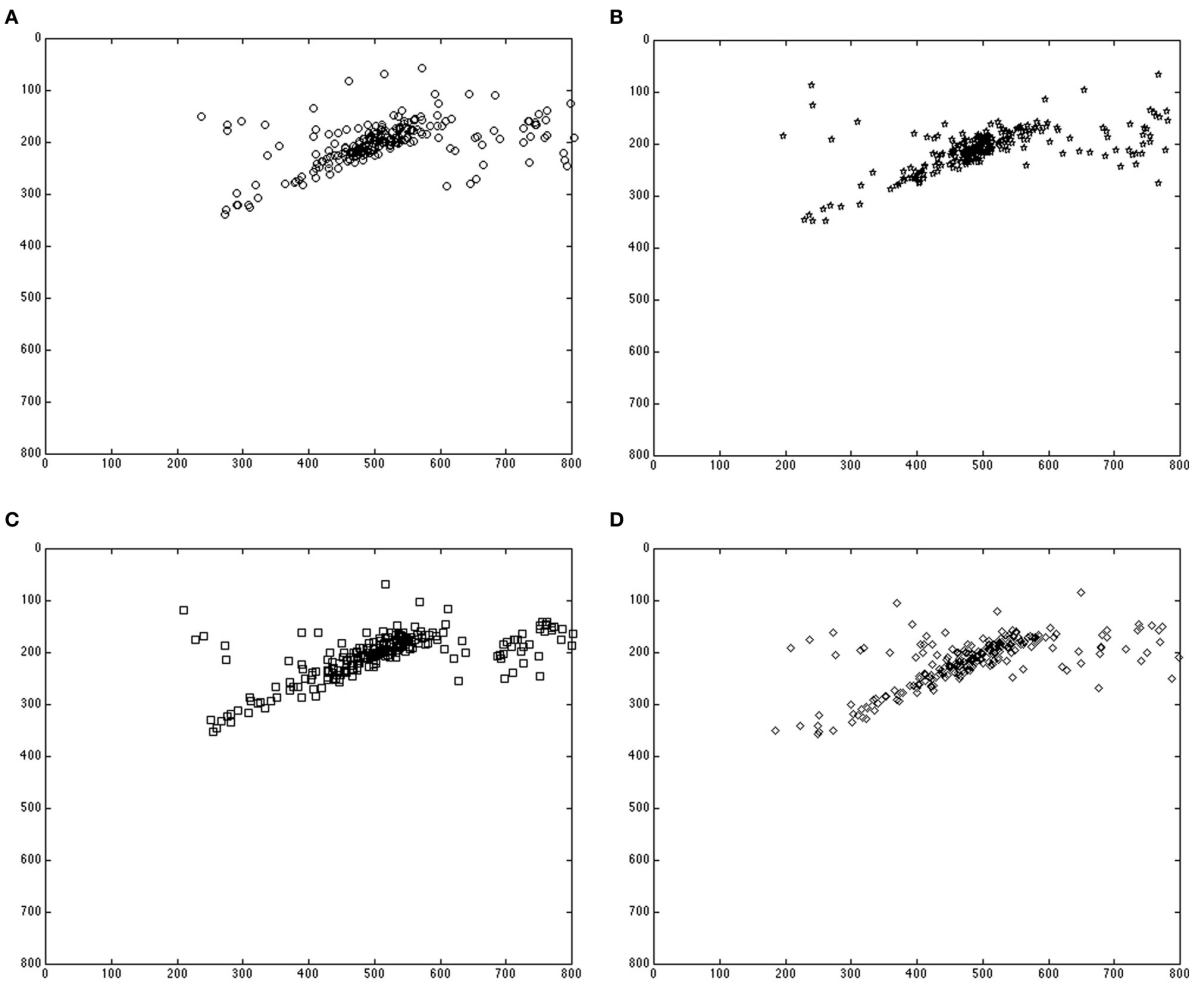
**Drop locations in response to simple past, recent past targets **(A)**; past progressive, recent past targets **(B)**; simple past, distant past targets **(C)**; and past progressive, distant past targets **(D)****.

First, we investigated the final placement of the character in each scene, precisely, where it was drop-clicked. We examined the x- and y-coordinates of the drop locations separately. In the x-coordinates, there was no significant interaction between aspect and temporal distance, nor was there a main effect of the temporal distance. However, there was a main effect of aspect, with the x-coordinates of the drop locations in response to simple past sentences being farther to the right (or closer to the destination, i.e., location of completed action) than those in response to past progressive sentences, *F*_(1, 60)_ = 12.47, *p* < 0.01. In the y-coordinates, there was no interaction of aspect and temporal distance. There was, however, a main effect of aspect, with the y-coordinates of the drop locations in response to listening to past progressive sentences being lower on the screen (closer to location of ongoing action) than those of the simple past sentences, *F*_(1, 60)_ = 10.26, *p* < 0.01. There was also a main effect of temporal distance, with the y-coordinates of the drop locations in response to listening to recent past descriptions being lower on the screen (again, closer to the location of ongoing action) than those of the distant past descriptions, *F*_(1, 60)_ = 4.31, *p* = 04, as shown in Figure 2.

These data are consistent with our earlier explorations of aspect using mouse-tracking. Specifically, aspect differentially influenced the final placement of the character, with an additive influence of temporal distance. When participants listened to past progressive sentences, they placed characters farther from the destination, or, closer to the location of ongoing action. When they listened to simple past sentences, they placed the character closer to the destination, namely, the location of completed action.

#### Spatial differences

**Figure 3:**
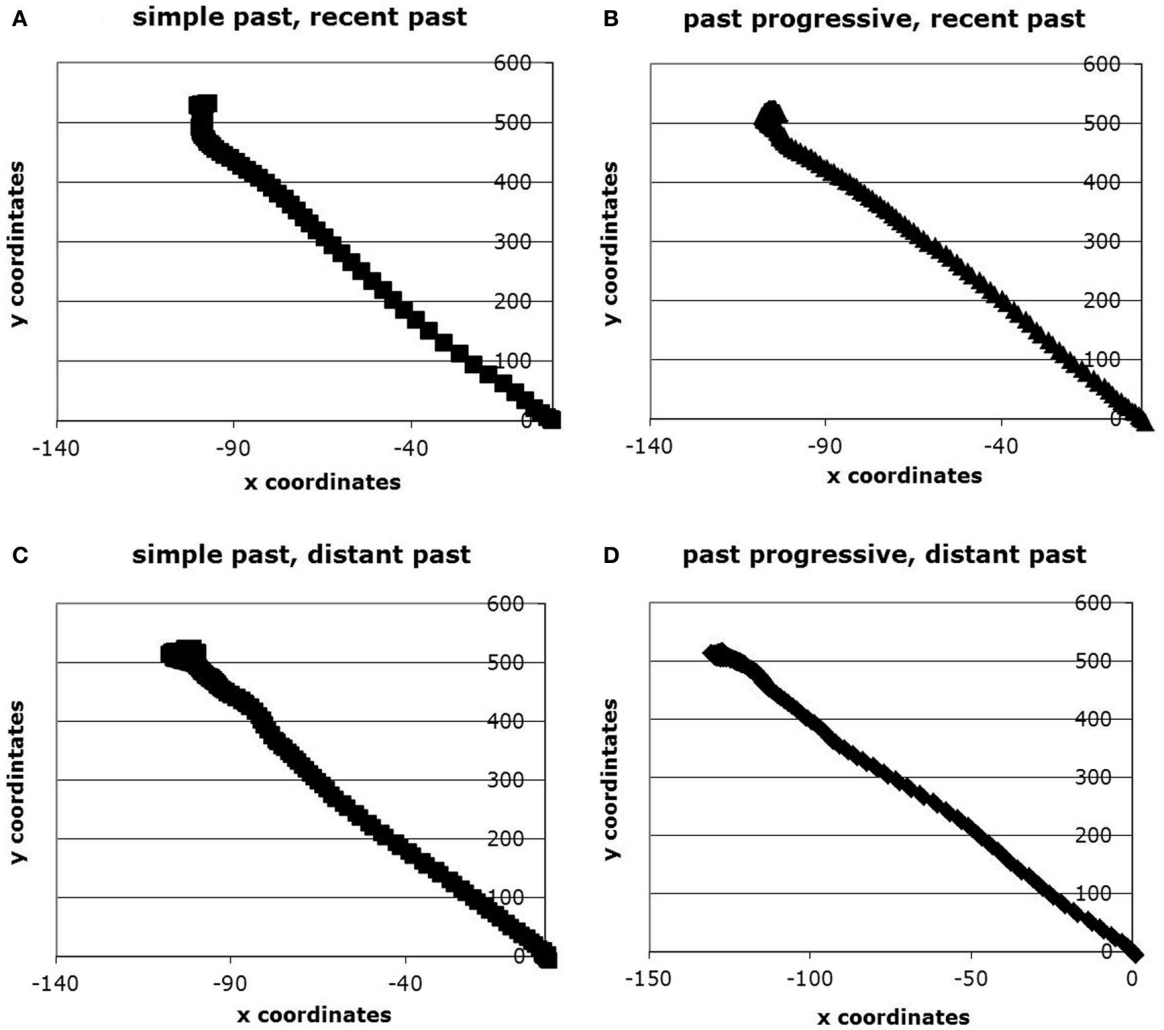
**Average time-normalized trajectories produced in response to simple past, recent past targets **(A)**; past progressive, recent past targets **(B)**; simple past, distant past targets **(C)**; and past progressive (PP) distant past targets **(D)****.

Figure 3 shows the average time-normalized trajectories in each of the four conditions. Since the [0, 0] x,y starting position is near the bottom center of the screen, leftward movements naturally take on negative *x*-values, and upward movements naturally take on positive *y*-values. Panel **(A)** shows the average time-normalized trajectory produced in response to sentences that included simple past recent past; panel **(B)** shows progressive recent past; panel **(C)** shows simple distant past; and panel **(D)** shows progressive distant past. Visual inspection shows differences among these four conditions, especially in the case of panel **(D)**, the past progressive, distant past targets. The averaged trajectory in panel **(D)** stretches leftward to an x-pixel value beyond −130, whereas the other conditions only reach to about −105.

To begin to statistically assess online aspectual differences, we looked at spatial differences between the average trajectories elicited in response to each of our conditions. To determine whether these averaged trajectories significantly diverged from each other, we time-normalized the trajectories and conducted a series of *t*-tests at each of the 101 time-steps. These analyses were conducted separately on the x- and the y-coordinates at each of the 101 time-steps to compare spatial differences across participants and across conditions. To avoid the increased probability of a Type-1 error associated with multiple *t*-tests, and in keeping with Bootstrap simulations of such multiple *t*-tests on mouse trajectories (see Dale et al., 2007), an observed divergence was not considered significant unless differences between the coordinates elicited *p*-values less than 0.05 for at least eight consecutive time-steps.

In the x-coordinates, there was no interaction or main effect of temporal distance. However, there was a main effect of aspect in the x-coordinates between time-steps 45 and 101. The past progressive average trajectory diverged away from the simple past average trajectory and toward the path in the visual display, suggesting that the average past progressive trajectory was closer to the path on the screen, which is the location of the ongoing action. In the y-coordinates, there was no significant interaction, but there was a main effect of aspect between time-steps 54 and 101. Again, we observe the average past progressive trajectory adhered more closely to the path than did simple past average trajectories. There was also a main effect of temporal distance from time-steps 69–101, with recent past descriptions adhering more closely to the path than distant past description trajectories. Numerous studies have demonstrated that the continuous movement of a computer-mouse (or continuous movement of a hand) provides a moment-by-moment index of where visual attention is being deployed in the display (e.g., Song and Nakayama, 2006, 2009; Spivey et al., 2010). Therefore, it appears that past progressive sentences may have drawn attention to the location of the ongoing action, namely to the path, and that the simple past sentences may have drawn attention to the location of the completed action. Additively, recent temporal information may also have encouraged greater attention to the path itself.

### Movement durations

**Figure 4:**
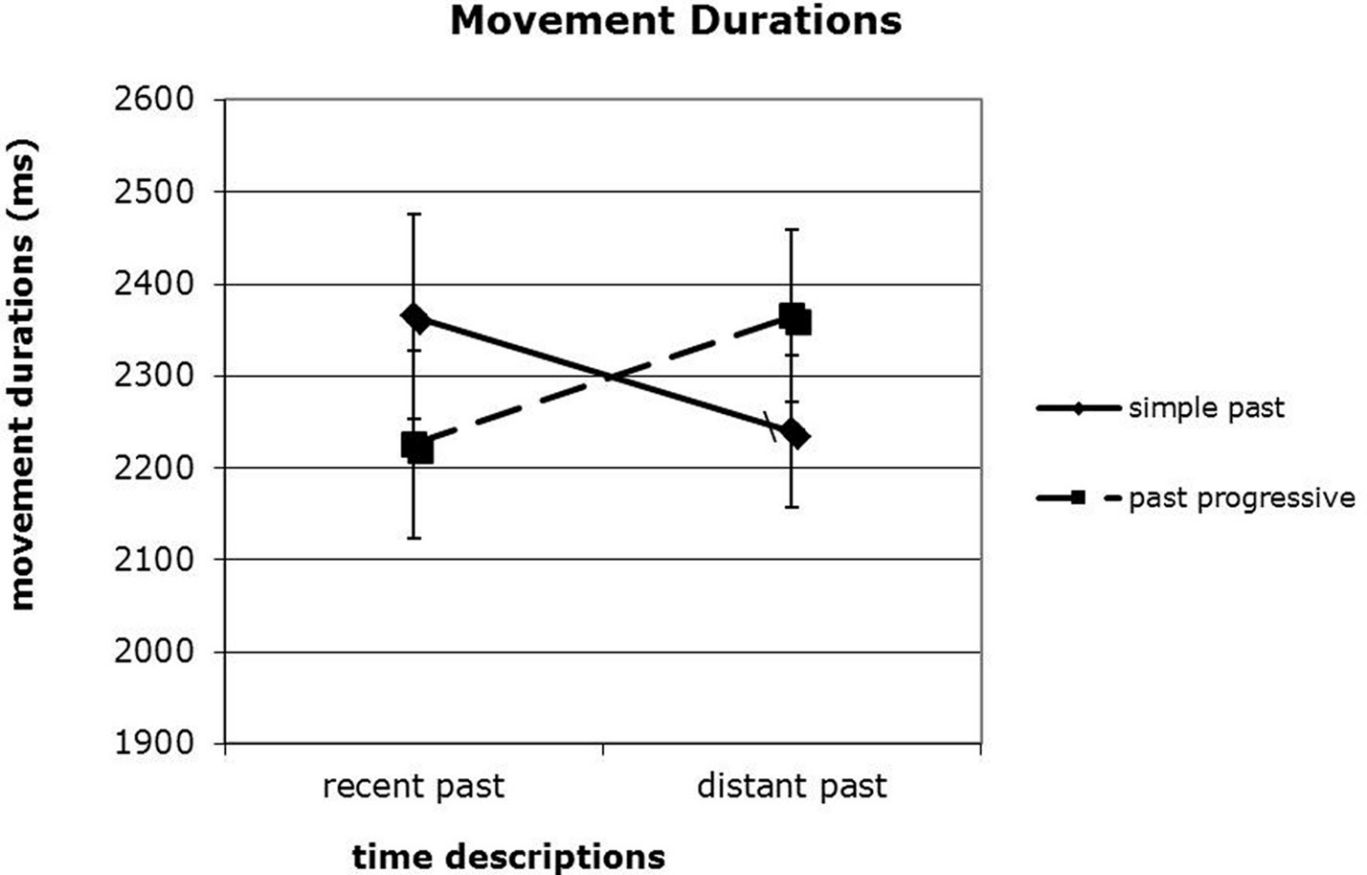
**Movement duration differences**.

Finally, we examined movement durations. Movement durations measured the time that it took participants to move the character from its departure position (grab-click) at the to its destination position (drop-click). This measurement was not merely a reaction time because it did not include the movement onset latency. Before we examined the movement durations, individual trials that exceeded 5.5 s (more than 2 standard deviations from the overall mean) were removed (less than 9% of the data). Notably, the average length of the movement trajectories was approximately equal across all conditions. As shown in Figure 3, and discussed above, the past progressive condition tended to produce trajectories that extended to an endpoint about 25 pixels further along the x-axis, whereas the simple past condition tended to produce trajectories that extended to an endpoint about 25 pixels further along the y-axis. Therefore, with these trajectories extending about the same overall length, comparing the durations of them is a fair test of the speed and fluidity with which the action took place. Therefore, comparing the durations of these movements is a useful test of the speed and fluidity with which the action took place. Analysis of Variance of movement durations revealed no main effects, but did reveal a significant interaction of temporal information and aspect, *F*_(1, 60)_ = 4.63, *p* < 0.05, as shown in Figure 4. When the time frame was distant (i.e., “Last year”), movement durations in response to simple past sentences took less time (*M* = 2240.48, *SD* = 652.49) than those in response to past progressive sentences (*M* = 2365.62, *SD* = 735.35). However, when the time frame was recent (i.e., “Yesterday”), the pattern reversed. In that case, movement durations in response to simple past sentences took longer (*M* = 2365.86, *SD* = 869.65) than those produced in response to past progressive sentences (*M* = 2226.34, *SD* = 804.11). This interaction could have been driven by a variety of factors. Compatibility of aspect and temporal distance is one possible explanation. The pairing of simple past and distant past could have resulted in relatively quick, smooth movements. The simple past is associated with a snapshot interpretation or prominent end state (and not the ongoing nature) of an event, which is consistent with the distant past, i.e., too “far” to conceptualize in any detailed fashion. Similarly, the past progressive highlights the ongoing nature of an event, which is consistent with recent past, i.e., ongoing nature is highlighted because it has just happened. And pairings that were less compatible, i.e., simple past and recent past or with past progressive and distant past, could have resulted in longer movement durations.

It is important to stress again that these movement durations are not simply reaction time measures. Rather, they reflect time spent *moving* the character, not total time spent responding to the stimulus. Therefore, it could be that while the hand-and-mouse were in the process of executing the placement of the character, these temporal characteristics of the perceptual simulation were spreading out into that motor movement itself. Thus, while the past progressive placed emphasis on the ongoing intermediate stages of the event (as though it were still happening), the context placed the event in the distant past, resulting in a mismatch that manifested itself as slower movement of the character. Similarly, when the simple past condition induced an emphasis on the static completed state of the event (as though it was in the distant past), but the context placed the event in the recent past, this mismatch again resulted in longer movement durations. Commensurate with earlier investigations of aspect, past progressive processing appears to correspond to diffuse, intermediate stages of an event, and simple past processing, with the end state (Madden and Zwann, 2003).

## Discussion

The results reported here provide new insights into how information about grammatical aspect and temporal distance interact to shape perceptual simulations in the understanding of event descriptions. First, in analyzing drop locations, we found that aspectual information (simple past vs. past progressive) influenced where participants placed the character in the scene, with an additive influence of temporal context (distant vs. recent past). When participants heard recent past progressive descriptions, such as “Yesterday David was walking to the university,” they placed the character closer to the location of ongoing action (on the path, where the character did the walking) and farther from the destination, than they did when they listened to distant simple past event descriptions, such as “Last year David walked to the university.” Second, the spatial differences analysis showed a consistent pattern: past progressive sentences and, additively, recent past temporal information, appeared to draw attention to the location associated with ongoing action, while simple past sentences, to the location associated with completed action. Finally, our movement duration data revealed a full interaction of aspect and temporal context.

The results with final placement data and with spatial differences data show the expected findings, and provide compelling support for the effects of aspect and temporal context, yet the interaction in the movement duration data is not what one might have initially expected. Based on findings with the other measures, the straightforward prediction for the movement duration data would have been for a main effect of aspect (where the past progressive would induce longer and slower movement durations that practically “act out” the emphasis on the ongoingness of the event), and a main effect of temporal distance (where recent past would also induce longer and slower movement durations resulting from the simulated recency and availability of the event and its temporal details). However, instead of finding these two main effects, we observed a surprisingly well-balanced crossover interaction of the two factors. Given the support for perceptual simulations in the other measures, and with previous versions of these sentences, the lack of these two main effects is puzzling, and may be due to the greater complexity of the sentences resulting from adding temporal context. If we had indeed found such a pair of main effects, some concern might have arisen about the comprehension of the stimuli in the distant past context and the simple past condition, e.g., “Last year David walked to the university.” Note that in English, the distant simple past has an inherent ambiguity: a distant simple past event can be construed as iterative (as if to mean, “All last year David regularly walked to the university.”), or as a one-time event (as if to mean, “Last year for his first and only time, David walked to the university.”). Based on the results of this single experiment alone, it is not possible to determine how participants interpreted some of our distant simple past verbal stimuli. Some the distant simple past items may have been interpreted as iterative. Future research with experiments that include a range of time frames and a variety of verb types will be informative, and help obtain a better picture of how processing unfolds in time.

When the interaction between aspect and temporal distance in the movement duration data is examined on its own, the result suggests a resonance account where linguistic devices that share semantic properties tend to induce smooth, fast, and unhindered processing (not unlike phenomena observed in the action-sentence compatibility effect; Glenberg and Kaschak, 2002). For example, perfective aspect (simple past in English) and a distant past context both tend to mentally package the event as an atomic unit whose emphasis is on the completed end-state, so they are compatible with one another. Thus, when distant past and simple past are paired, the completion of the simple past event description resonates with the distant past description, and, hence, the movement trajectory is fast, smooth, and brief. By contrast, imperfective aspect (past progressive in English) and a recent past context both mentally represent the event as a drawn out process whose intervening temporal details are available and emphasized, so they are compatible with one another. Therefore, when the event is in the recent past, the ongoingness of the past progressive resonates with that temporal description, so again response movements are fast, smooth, and brief. However, when the pairings do *not* resonate with each other, as in either simple past with recent past or past progressive with distant past, the two do not match in the level and type of detail invoked, and consequently, the movement trajectories are not as smooth or fast. In future work, it will be useful and informative to consider how natural or familiar these pairings are, in particular, how frequent they are across a range of contexts. Some forms may occur more often and possibly be more natural to process than others. Statements such as “Last year David was walking to the university” certainly occur in everyday English, but they may be less common than statements such as “Yesterday David was walking to the university.” It is possible that naturalness of these pairings influenced our results.

These data add to our understanding of how grammatical aspect influences language comprehension, especially with various types of temporal information. Our results expand previous research on the role of aspect in event descriptions, including investigations with mouse-tracking (Anderson et al., 2008, 2010), narrative comprehension (Magliano and Schleich, 2000; Madden and Zwann, 2003), surveys (Fausey and Matlock, 2010; Matlock, 2011), language production in natural discourse (Matlock et al., 2012), and offline spatial judgment tasks (Matlock et al., 2007). The consistent pattern that emerges from these varied methodologies is that grammatical aspect systematically influences perceptual simulations that drive language comprehension, for instance, enhancing or diminishing certain properties of events.

These results also contribute to research on the linguistic connection between time and space. In particular, they complement previous research on space as a metaphor for time. People often describe time in terms of physical space (Clark, 1973; Traugott, 1978; Alverson, 1994). Importantly, this relationship tends to be asymmetrical: people use space to talk about time far more often than they use time to talk about space (Lakoff and Johnson, 1980, 1999). Even when people are asked to make non-linguistic judgments about time, they recruit spatial metaphors (Cassasanto and Boroditsky, 2007), suggesting that understanding time in terms of space is not simply a matter of linguistic convention. More importantly, in understanding events, people understand naturally think about and communicate about “where” things happen in time relative to the time of reporting, for instance, near past or distant past (Trope and Liberman, 2003, 2010). So, events in the recent past are processed with rich detail, and events of the more distant past are processed with less detail (Liberman and Trope, 2008).

Many questions remain about the processing of grammatical aspect, and certainly there are alternative explanations. For example, past progressive descriptions may somehow be more effortful to comprehend than simple past sentences. People might think about actions in a more engaged, moment-by-moment way with past progressive descriptions than they do with simple past descriptions. Previous research is also unclear on this point. Madden and Zwann (2003), for instance, found that participants took more time to process progressive sentences, possibly because they were more difficult to comprehend. Differences in processing various forms of aspect may also arise because of verb semantics. Careful study of telicity, person, voice, and other semantic dimensions of verb meaning need to be given careful attention in the study of aspect (see Matlock, 2011; Croft, 2012). This could help clarify issues that we were unable to address, including iterative interpretation with sentences, such as “David walked to the university last year.” The focus here was on literal translational motion verbs (i.e., verbs that convey contiguous movement from one point in space to another).

Our findings have implications for research on event understanding. They show how subtle differences in aspect alone can systematically influence motion events are conceptualized. They also provide new insights on how aspect influences thought about events in the near and distant past. They contribute to a growing body of research on how events are conceptualized differently depending on “where” they are relative to the time of reporting (e.g., Trope and Liberman, 2003, 2010; Liberman and Trope, 2008). The work helps expand a new, exciting line of research on how grammatical information can influence construal of events (see, Kaup et al., 2010, for instance, for a study on how German speakers process sentences with adjectives and adjectival passives). Last, our results provide evidence to support cognitive linguists' claims about how grammar has meaning rooted in our embodied experience (Langacker, 1987; Lakoff and Johnson, 1999; Talmy, 2000).

This research resonates with embodied cognition work on perceptual simulation and language understanding (Barsalou, 1999). It is consistent with the methodological advances of Balota and Abrams (1995) by providing new evidence from the temporal dynamics of a response after the it has been initiated, and by demonstrating that the motor system is not a robot-like automaton triggered by completed cognitive processes. Rather, motor processes co-exist with cognitive processes during perceptual/cognitive tasks (e.g., Balota and Abrams, 1995; Gold and Shadlen, 2000; Spivey et al., 2005). This work also aligns with our understanding of how mental models and visual information are coordinated in motor output. Similar to the way understanding spatial events is created and observed through tracking eye movements (Spivey and Geng, 2001; Richardson and Matlock, 2007), this work shows that event understanding varies as a function of changes in aspect and temporal distance. Our results add to the emerging pattern of data that suggest that differences underlying perceptual simulations, resulting in these differences in the dynamics of a motor response, may account for observed processing differences in comprehending sentences that use different aspectual forms. This means that perceptual simulations behave in predictable ways, even when it comes to grammatical aspect.

### Conflict of interest statement

The authors declare that the research was conducted in the absence of any commercial or financial relationships that could be construed as a potential conflict of interest.
